# Improving detection of JC virus by ultrafiltration of cerebrospinal fluid before polymerase chain reaction for the diagnosis of progressive multifocal leukoencephalopathy

**DOI:** 10.1186/s12883-019-1476-2

**Published:** 2019-10-25

**Authors:** Kazuo Nakamichi, Michi Kawamoto, Junko Ishii, Masayuki Saijo

**Affiliations:** 1 Department of Virology 1, National Institute of Infectious Diseases, Toyama 1-23-1, Shinjuku-ku, Tokyo, 162-8640 Japan; 2 Department of Neurology, Kobe City Medical Center General Hospital, 2-1-1, Minatojimaminamimachi, Chuo-ku, Kobe City, Hyogo 650-0047 Japan

**Keywords:** Cerebrospinal fluid, JC virus, Progressive multifocal leukoencephalopathy, Real-time PCR testing, Ultrafiltration

## Abstract

**Background:**

Progressive multifocal leukoencephalopathy (PML) is a demyelinating disorder caused by JC virus (JCV). Although detecting JCV DNA in the cerebrospinal fluid (CSF) by real-time polymerase chain reaction (PCR) is useful, diagnosis is difficult when JCV concentrations are low. We therefore aimed to lower the detection limit of real-time PCR testing by enriching JCV in the CSF via ultrafiltration.

**Methods:**

Virus suspensions and CSF specimens from 20 untreated patients with suspected PML were collected and total DNAs were extracted. The JCV large T gene was detected by quantitative real-time PCR under condition with and without prior centrifugal ultrafiltration.

**Results:**

The JCV DNA was reliably detected to a lower limit of 10 copies/mL of virus suspension by real-time PCR with ultrafiltration. When using this method, the quantity of JCV DNA per PCR reaction increased 3.2- to 8.7-fold compared with the standard procedure. Seven patients were positive for JCV when using the standard procedure, and an additional patient was positive when using ultrafiltration. All JCV-positive patients had neurological features and magnetic resonance imaging findings compatible with PML.

**Conclusions:**

The detection limit of JCV DNA by real-time PCR can be lowered by viral enrichment using ultrafiltration. Our simple protocol offers a valuable tool for PML diagnosis when extremely low copy numbers of JCV are released into the CSF or when brain biopsy is not feasible.

## Background

Progressive multifocal leukoencephalopathy (PML) is a rare and often fatal demyelinating disorder caused by JC virus (JCV), a double-stranded DNA virus of the family *Polyomaviridae* [1–3]. JCV is ubiquitous in human populations, with 50–80% of adults reported to be serologically positive [1, 4, 5]. The initial JCV infection is thought to occur asymptomatically during childhood, when the virus establishes persistent infection in the kidney and urinary tract [6]. JCV then persists or is latent in other tissues, such as the spleen, tonsils, and bone marrow [3, 5].

Under conditions of immunosuppression or altered trafficking of immune cells, JCV reactivates and lytically infects myelin-producing oligodendrocytes, causing demyelination [3, 5, 7]. It is known that PML develops in patients immunosuppressed due to human immunodeficiency virus (HIV) infection, lymphoproliferative disease, transplantation, or chemotherapy for malignancy [2, 8, 9]. Moreover, PML has been increasingly diagnosed in patients receiving immunosuppressive or immunomodulatory therapies for autoimmune disorders, including multiple sclerosis, systemic lupus erythematosus, and rheumatoid arthritis [1, 2, 8, 10, 11].

The detection of JCV DNA by polymerase chain reaction (PCR) of cerebrospinal fluid (CSF) is a reliable and less-invasive diagnostic marker of PML, particularly when combined with magnetic resonance imaging (MRI) findings and neurologic symptoms [12]. Therefore, CSF testing by quantitative real-time PCR has become the diagnostic standard [12, 13]. Prior to the development of an ultrasensitive PCR assay for JCV DNA, the sensitivity of this method was reported to be about 74% [14]; by contrast, ultrasensitive PCR techniques have been shown to have sensitivities exceeding 95% [12]. Although real-time PCR testing of CSF specimens is useful, differentiating positive and negative results can be difficult when extremely faint amplification signals are present (e.g., few copies per reaction). However, DNA extraction procedures from CSF, either by manual or automated methods, are limited by the concentration and sample volume. If viral particles from larger volumes of CSF can be concentrated, JCV DNA could be more reliably detected by PCR.

JCV is occasionally excreted in the urine of healthy individuals and is stable in the aquatic environments. Thus, the virus is sometimes used as a biological indicator of water quality and pollution [15–19]. In environmental studies, JCV particles in water samples are concentrated by ultrafiltration, and this technique could be valid for application with JCV virions in clinical CSF samples prior to PCR testing. The present study was undertaken to establish and validate quantitative real-time PCR assaying of JCV in CSF specimens following centrifugal ultrafiltration.

## Methods

### CSF specimens and clinical data

The study was approved by the medical research ethics committee for the use of human subjects at the National Institute of Infectious Diseases (NIID), Tokyo, Japan (approval numbers 667 and 964). It was performed in accordance with the ethical standards of the Declaration of Helsinki. Written informed consent was obtained from patients or their families.

In Japan, real-time PCR testing of CSF specimens for JCV DNA is routinely performed at the Department of Virology 1, NIID [9, 20–26]. For this study, CSF specimens were collected by lumbar puncture from 21 individuals. Of these, one patient had previously been diagnosed with PML. The JCV-positive CSF obtained during follow-up of this patient was diluted with Dulbecco’s phosphate-buffered saline (DPBS, sterile-filtered, calcium- and magnesium-free, Thermo Fisher Scientific Inc., Waltham, MA) and used as a standard virus suspension to compare the performance of our PCR assay. The remaining 20 patients had suspected PML based on neurological symptoms and/or brain MRI findings. The initial CSF specimens were subjected to real-time PCR testing to detect JCV DNA with and without undergoing ultrafiltration.

All CSF samples were frozen at the source hospitals without centrifugation and were transported to the NIID in solid carbon dioxide, at which point they were stored at − 80 °C until further analyses. Anonymous clinical data were obtained from physicians, using standardized questionnaires. The data were analyzed with respect to age, gender, underlying disease, neurologic symptoms, and brain MRI findings.

### CSF ultrafiltration

**Fig. 1:**
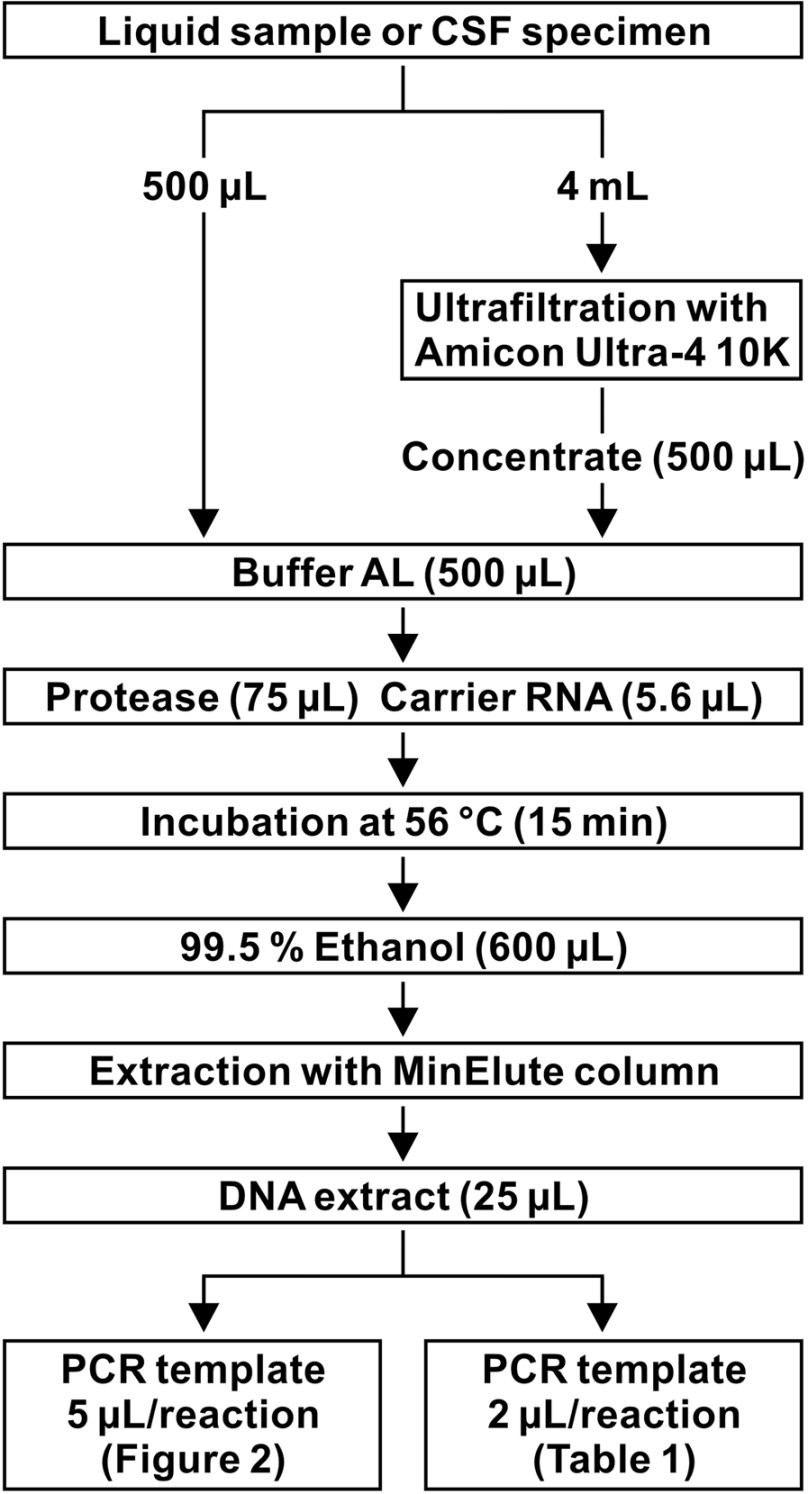
Schematic diagram of the handling procedures for ultrafiltration and DNA extraction. Standard virus suspensions or CSF specimens were left untreated or concentrated with ultrafiltration. The samples were lysed with Buffer AL, and total DNA was extracted using a QIAamp MinElute Virus Spin Kit

JCV particles in fluid samples were concentrated using an Amicon Ultra-4 10 K centrifugal filter device with a 10 kDa molecular weight cut-off membrane (EMD Millipore Corporation. Billerica, MA; hereafter called filter device). Centrifugation was executed with an Eppendorf 5804R centrifuge and an A-4-44 swing rotor (Eppendorf, Hamburg, Germany). The filter device, contained in a 15-mL centrifuge tube (BD Biosciences, Franklin Lakes, NJ), was spun in a centrifuge bucket sealed with an aerosol-tight cap (Eppendorf) for the use of clinical specimens that may contain potentially infectious agents. In addition, all centrifuge equipment (e.g., swing rotor, buckets, and sealing caps) were routinely irradiated with ultraviolet light to prevent DNA contamination before and after examination. The handling procedures are summarized in Fig. 1.

Frozen specimens were thawed at 37 °C in a block incubator (DTU-Neo; Taitec, Saitama, Japan) until the ice had just melted, before being mixed and temporarily stored in a refrigerator. To wash glycerol from the ultrafiltration membranes, 4 mL of DPBS was added to the filter device and spun at 4000×*g* for 4 min at room temperature. The filtrate in the centrifuge tube and any residual liquid in the filter device were discarded, and these wash steps were repeated to a total of three times. Next, a 4-mL CSF sample was added to the filter device in a fresh 15-mL centrifuge tube and was centrifuged at 4000×*g* for 8–10 min at 25 °C until the concentrate volume was slightly less than 500 μL. The concentrate was stirred by flushing with a pipette at least five times inside the filter device, and its volume was accurately adjusted to 500 μL with DPBS. The concentrate was mixed with 500 μL of lysis buffer containing guanidine hydrochloride (Buffer AL; Qiagen, Venlo, Netherlands) and subjected to nucleic acid extraction and real-time PCR analysis.

### DNA extraction

Total DNAs were extracted from CSF samples using a QIAamp MinElute Virus Spin Kit (Qiagen), according to the manufacturer’s protocol with slight modification. This kit is designed for DNA/RNA extraction with a starting sample volume of 200 μL. To obtain more concentrated extracts from larger samples, we used 500 μL of original or ultrafiltrated CSF samples for DNA extraction and used the manufacturer’s protocol for a similar extraction kit (QIAamp MinElute Virus Vacuum Handbook 3rd edition; Qiagen). Briefly, the CSF sample was mixed with 500 μL of Buffer AL, 75 μL of Qiagen Protease (1.07 Anson units/mL; Qiagen), and 5.6 μL of carrier RNA (1 μg/μL; Qiagen). After incubation at 56 °C for 15 min in the block incubator, 600 μL of 99.5% ethanol was added to the lysate, and the resulting mixture was passed through the MinElute column for centrifugation at 6000×*g* for 1 min, three times. The MinElute column was washed, 30 μL of distilled water (UltraPure DNase/RNase-Free Distilled Water; Thermo Fisher Scientific Inc.) was applied, and 25 μL of DNA extract was collected. DNA samples were then subjected to real-time PCR assay.

### Real-time PCR assay

Quantitative real-time PCR assay targeting the JCV large T gene was carried out, as described in the earlier reports [9, 20–26]. To confirm the detection of JCV genomic DNA, real-time PCR for the JCV viral protein 1 (*VP1*) gene was conducted [9]. In addition, sample contamination with standard DNA plasmid was monitored by real-time PCR that detected standard DNA but not the JCV genome [9]. These PCR assays were capable of detecting at least four copies of the target DNA per reaction [9].

### Statistics

Differences in the copy numbers of JCV DNA in CSF specimens were compared by parametric analyses using the paired Student’s *t*-test. All *P*-values less than 0.05 were considered statistically significant.

## Results

### Real-time PCR detection of JCV DNA in suspensions concentrated by ultrafiltration

The first analyses examined whether the detection limit of real-time PCR targeting JCV DNA could be lowered by ultrafiltration. Standard virus suspensions containing known copy numbers of JCV were left untreated or were concentrated by ultrafiltration, and total DNA was extracted for real-time PCR assay, targeting the JCV large T gene. In these analyses, JCV was diluted with DPBS but not with JCV-negative CSF because the large amount of CSF specimen could not be obtained.

**Fig. 2:**
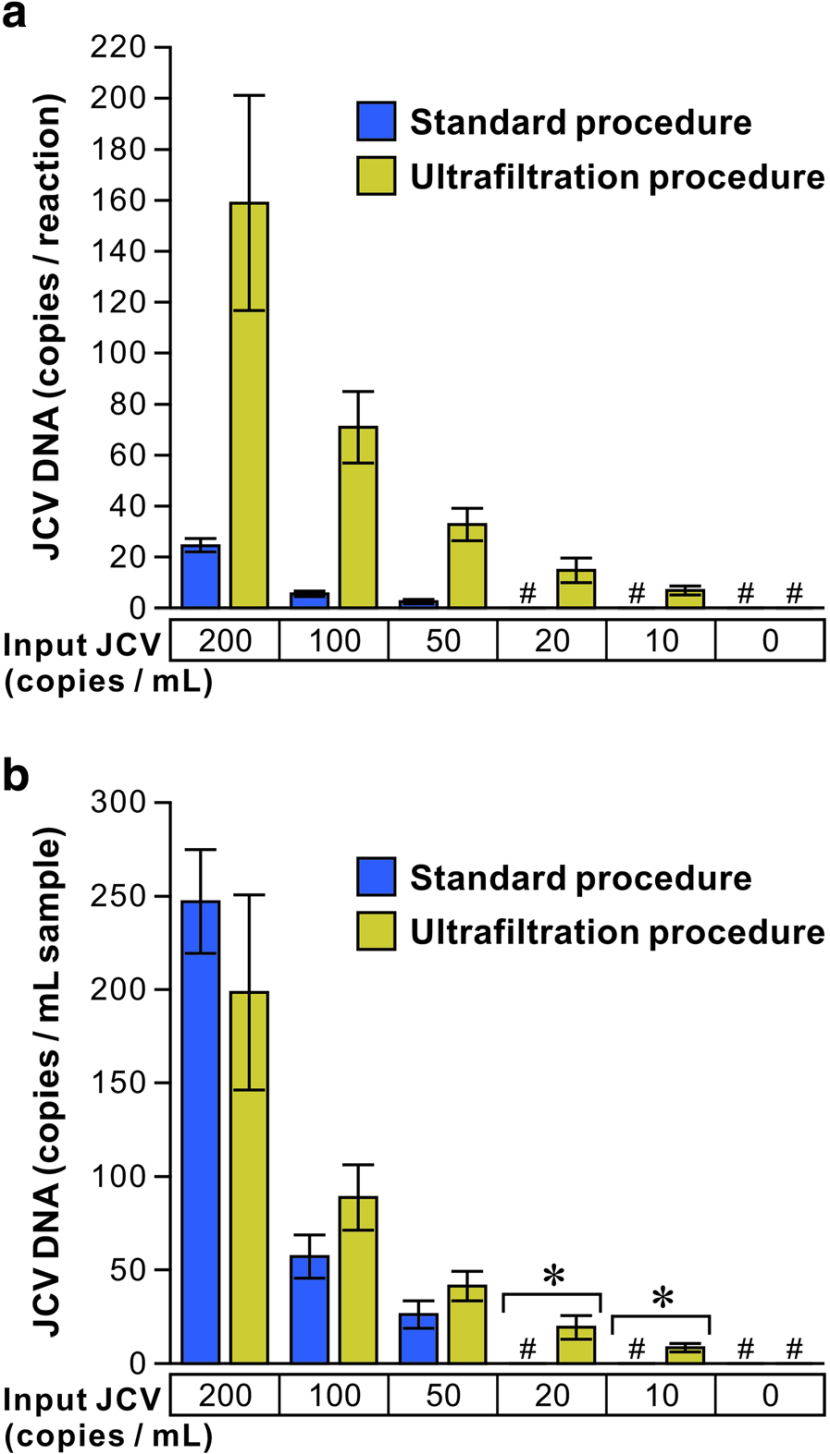
Real-time PCR quantitation of JCV DNA in virus suspensions following ultrafiltration. The standard virus suspensions, which contain the indicated amounts of JCV were left untreated (standard procedure) or concentrated from 4 mL to 500 μL in volume by using the ultrafiltration device (ultrafiltration procedure) as described in the legend of Fig. 1. Total 25-μL DNAs were extracted from a 500-μL sample, and the copy numbers of the JCV DNA per PCR reaction (5 μL template) were determined (**a**). The copy numbers of JCV DNA per mL were calculated with respect to total volume of DNA extracts (5-fold) and the amounts of test samples (either 2-fold for standard procedure or 0.25-fold for ultrafiltration procedure) (**b**). Data are shown as means ± standard errors of the means from four separate experiments. Sharp symbols indicate that JCV DNA was under the detection level. Statistically significant differences in the copy numbers of JCV DNA following the standard and ultrafiltration procedures are indicated by asterisks (*p* < 0.05). Statistical analyses were performed only on the data shown in panel B because the initial volume and concentration ratio for the data of panel A are not equal between each procedure

The copy numbers (copies/reaction) of JCV DNA in 5 μL of DNA extract measured by real-time PCR are shown in Fig. 2a. When the PCR templates were prepared from 500 μL of the standard virus suspensions without ultrafiltration (“the standard procedure”), JCV DNA was detected in samples containing at least 50 viral genome copies per mL (copies/mL), and no amplification signal was found in the presence of 20 or 10 copies/mL (Fig. 2a). By contrast, when total DNAs were extracted from the standard virus suspensions concentrated from 4 mL to 500 μL by ultrafiltration (“the ultrafiltration procedure”), the JCV DNA could be detected down to 10 copies/mL. The absolute copy numbers of JCV DNA in the standard virus suspensions at each dilution step are shown in Fig. 2b, corrected for the concentration ratio. The copy numbers of JCV DNA in the standard virus suspension were similar with or without ultrafiltration in a range from 200 to 50 copies/mL, and there were no statistically significant differences between groups. These data suggest that the JCV DNA detection limit of PCR can be lowered by ultrafiltration, without affecting quantitative measurement quality.

### Real-time PCR testing after ultrafiltration of JCV DNA in CSF

To assess the performance of real-time PCR examination of JCV after ultrafiltration, CSF specimens from 20 patients suspected of having PML were subjected to DNA extraction with or without ultrafiltration. In these analyses, a small amount (2 μL) of DNA was used for each reaction because the CSF specimen volumes were limited. The results of quantitative PCR testing for the samples are summarized in Table 1. Seven CSF samples (patients 1–7) were positive for JCV DNA in the standard procedure. However, very low copies of viral genome (7–20 copies/reaction) were detected in the DNA extracts of three JCV-positive samples (patients 5–7). Because only a faint signal was observed in one of three independent PCR measurements, the CSF specimen from patient 8 was judged negative for JCV in the standard procedure. However, when CSF specimens were concentrated by ultrafiltration, the amounts of JCV DNA in each PCR reaction increased 4.3- to 8.7-fold, and patient 8 was found to be positive for JCV. The PCR results were verified by PCR targeting of *VP1*, and no contamination was observed as judged by the standard DNA-specific PCR. These data indicate that low copy numbers of JCV DNA in CSF specimens can become certainly detectable after ultrafiltration.

**Table 1:** PCR detection of JCV DNA in CSF specimens from patients suspected of having PML

	Standard procedure			Ultrafiltration procedure^a^		
Patient	Positive reaction^b^	Copies / reaction^c^	Copies/mL CSF^d^	Positive reaction	Copies / reaction^e^	Copies / mL CSF^f^
1	3/3	207,033	5,175,825	3/3	1,030,133	3,219,166
2	3/3	766	19,150	3/3	2433	7603
3	3/3	321	8025	3/3	1383	4322
4	3/3	123	3075	3/3	568	1775
5	3/3	20	500	3/3	110	344
6	3/3	12	300	3/3	93	291
7	3/3	7	175	3/3	61	191
8	1/3	NA	NA	3/3	6	19
9	0/3	NA	NA	0/3	NA	NA
10	0/3	NA	NA	0/3	NA	NA
11	0/3	NA	NA	0/3	NA	NA
12	0/3	NA	NA	0/3	NA	NA
13	0/3	NA	NA	0/3	NA	NA
14	0/3	NA	NA	0/3	NA	NA
15	0/3	NA	NA	0/3	NA	NA
16	0/3	NA	NA	0/3	NA	NA
17	0/3	NA	NA	0/3	NA	NA
18	0/3	NA	NA	0/3	NA	NA
19	0/3	NA	NA	0/3	NA	NA
20	0/3	NA	NA	0/3	NA	NA

Abbreviations: *CSF* Cerebrospinal fluid, *JCV* JC virus, *NA* Not applicable, *PCR* Polymerase chain reaction, *PML* Progressive multifocal leukoencephalopathy

^a^Each CSF specimen was concentrated from 4 mL to 500 μL by ultrafiltration before DNA extraction

^b^The PCR was repeated three times

^c^The mean copy numbers of JCV DNA in 2-μL PCR template of total 25-μL DNA extract from 500-μL CSF were determined, when all three PCRs were positive

^d^The copy numbers of JCV DNA per mL were calculated with respect to total volume of DNA extracts (12.5-fold) and the amounts of test samples (2-fold)

^e^The mean copy numbers of JCV DNA in 2-μL PCR template of total 25-μL DNA extract from 4-mL CSF were determined, when all three PCRs were positive

^f^The copy numbers of JCV DNA per mL were calculated with respect to total volume of DNA extracts (12.5-fold) and the amounts of test samples (0.25-fold)

### Diagnostic evaluation based on the PCR results and clinical characteristics

A final set of analyses were carried out to evaluate the utility of ultrafiltration before real-time PCR testing of the CSF as an aid to clinical diagnosis. The backgrounds of patients who were positive or negative for JCV DNA in the real-time PCR assays after ultrafiltration are summarized in Table 2. The JCV-positive group (patients 1–8) and the JCV-negative group (patients 9–20) appeared comparable with respect to age and gender. However, all JCV-positive patients, except patient 3, had underlying diseases that may have affected their immunocompetence, such as hematological malignancy, autoimmune disorders, or HIV infection. Patient 3 had nephrotic syndrome and was treated with prednisolone. On the other hand, three of the 12 patients in the JCV-negative group (patients 13, 15, and 18) had no apparent underlying disease.

**Table 2:** Clinical background of patients with positive and negative CSF for JCV DNA

Patient	CSF JCV	Age (y)	Sex	Underlying disease	Immunosuppressive factor
1	Positive	88	F	Chronic lymphocytic leukemia	Unknown^a^
2	Positive	58	F	Systemic lupus erythematosus Lupus nephritis Sjögren’s syndrome	Prednisolone Methotrexate
3	Positive	59	F	Nephrotic syndrome	Prednisolone
4	Positive	36	F	Systemic lupus erythematosus	Prednisolone Cyclophosphamide
5	Positive	38	F	Systemic lupus erythematosus Lupus nephritis	Prednisolone Mycophenolate mofetil
6	Positive	42	M	HIV infection	AIDS
7	Positive	63	F	Multiple sclerosis	Fingolimod
8	Positive^b^	41	M	HIV infection	AIDS
9	Negative	70	F	Sarcoidosis	Prednisolone
10	Negative	34	M	Diffuse large B-cell lymphoma	Cytarabine Methotrexate Rituximab
11	Negative	65	M	Liver disease (transplantation)	Tacrolimus Mycophenolate mofetil
12	Negative	67	F	Drug-induced nephropathy	Cyclosporine
13	Negative	52	M	No disease	Unknown
14	Negative	81	F	Burkitt’s lymphoma	Chemotherapy (R-EPOCH) Methotrexate
15	Negative	72	M	No disease	Unknown
16	Negative	48	F	Multiple sclerosis	Glatiramer acetate
17	Negative	56	M	Adult T-cell leukemia/lymphoma	Thymoglobulin Chemotherapy (mLSG15)
18	Negative	53	M	No disease	Unknown
19	Negative	50	M	HIV infection	AIDS
20	Negative	53	F	Follicular lymphoma	Chemotherapy (R-CHOP) Methotrexate

Abbreviations: *AIDS* Acquired immunodeficiency syndrome, *CSF* Cerebrospinal fluid, *HIV* Human immunodeficiency virus, *JCV* JC virus, *mLSG15* modified Lymphoma Study Group 15, *R-EPOCH* Rituximab plus etoposide, prednisone, vincristine, cyclophosphamide, doxorubicin, *R-CHOP* Rituximab plus cyclophosphamide, doxorubicin, vincristine, and prednisone

^a^Patient received neither radiation therapy nor chemotherapy

^b^JCV DNA was detected in the ultrafiltrated CSF specimen

The distributions of brain lesions and neurologic symptoms in the JCV-positive and JCV-negative groups are shown in Table 3. Hyperintense lesions on T2/fluid-attenuated inversion recovery imaging were found mainly in the cerebral white matter, cerebellar white matter, and/or brain stems. Bilateral symmetric brain lesions were observed in more than 60% of the JCV-negative group (patients 12–14 and 17–20), but all patients in the JCV-positive group presented with either unilateral or bilateral asymmetric lesions. In addition, five patients in the JCV-positive group had multiple and severe neurological symptoms (patients 1, 3, 5, 6, and 8). All patients with CSF positive for JCV were finally diagnosed with PML.

**Table 3:** Distribution of brain lesions and neurologic symptoms in patients with JCV-positive and JCV-negative CSF samples

		MRI (T2/FLAIR) lesion (%)^a^					
Patient	JCV DNA	Cerebral white matter	Cerebellar white matter	Brain stem	Pattern	Symmetry	Neurologic symptom
1	Positive	+	–	–	Bilateral	Asymmetric	Quadriplegia, Consciousness disorder, Aphasia, Involuntary movement, Increased deep tendon reflex
2	Positive	–	+	–	Unilateral	Asymmetric	Cerebellar symptom
3	Positive	+	+	–	Bilateral	Asymmetric	Hemiplegia, Mental manifestation, Dysarthria, Dysphagia, Increased deep tendon reflex
4	Positive	–	+	–	Unilateral	Asymmetric	Cerebellar symptom
5	Positive	+	–	+	Bilateral	Asymmetric	Dysarthria, Sensory disturbance, Cerebellar symptom
6	Positive	+	+	+	Bilateral	Asymmetric	Consciousness disorder, Mental manifestation, Aphasia, Dysarthria, Dysphagia, Sensory disturbance, Involuntary movement
7	Positive	+	–	–	Bilateral	Asymmetric	Consciousness disorder, Aphasia
8	Positive^a^	+	+	+	Bilateral	Asymmetric	Hemiplegia, Consciousness disorder, Increased deep tendon reflex, Cerebellar symptom
9	Negative	+	–	–	Unilateral	Asymmetric	Hemiplegia, Consciousness disorder, Aphasia
10	Negative	+	–	–	Bilateral	Asymmetric	Increased deep tendon reflex
11	Negative	+	–	–	Unilateral	Asymmetric	Sensory disturbance
12	Negative	–	–	+	Bilateral	Symmetric	Sensory disturbance
13	Negative	+	–	–	Bilateral	Symmetric	Consciousness disorder, Mental manifestation
14	Negative	+	–	–	Bilateral	Symmetric	Consciousness disorder
15	Negative	+	–	–	Bilateral	Asymmetric	Consciousness disorder, Dysarthria
16	Negative	+	–	+	Bilateral	Asymmetric	Consciousness disorder, Sensory disturbance
17	Negative	+	+	–	Bilateral	Symmetric	Quadriplegia, Consciousness disorder
18	Negative	+	+	+	Bilateral	Symmetric	Dysarthria, Cerebellar symptom
19	Negative	+	+	+	Bilateral	Symmetric	Consciousness disorder, Dysarthria, Sensory disturbance, Increased deep tendon reflex, Cerebellar symptom
20	Negative	+	–	–	Bilateral	Symmetric	Consciousness disorder, visual disturbance, Dysarthria, Dysphagia, Sensory disturbance

Abbreviations: *CSF* Cerebrospinal fluid, *FLAIR* Fluid-attenuated inversion recovery, *JCV* JC virus, *MRI* Magnetic resonance imaging

^a^Plus indicates that MRI lesions were identified, while minus indicates that no lesions were found

^b^JCV DNA was detected in ultrafiltrated CSF specimen

## Discussion

The detection of JCV in the CSF is a key factor in the diagnosis of PML. Several reports, however, have demonstrated false-negative results by PCR testing in patients diagnosed with PML [27–29]. In these cases, JCV DNA and/or protein were detected in brain tissues on pathological analyses, despite negative CSF samples on standard PCR testing. We also recently presented a case in which PML was only diagnosed by brain biopsy [28]. In this case, the patient was not positive for JCV in the CSF because the real-time PCR amplification signal was too faint and unstable, probably due to the extremely low copy number of the target DNA. However, if brain biopsy had not been possible because the lesions were too deep, the patient had underlying medical conditions, or patient refused the procedure, CSF analysis might have been the only way to confirm JCV and therefore PML. Virion enrichment by ultrafiltration from a larger CSF volume can improve the sensitivity and reliability of PCR testing in this setting.

Given the size of viral particles, ultrafiltration membranes with large molecular cut-offs (e.g., 100 kDa) should be appropriate. However, in preliminary experiments, CSF specimens were too rapidly concentrated to a very small volume after ultrafiltration with these membranes, and it was difficult to handle the samples. In the current protocol, a centrifugal filter device with a 10 kDa molecular weight cut-off membrane was used for ultrafiltration of the CSF. Although the procedure was simple and easy to perform, it was unclear whether the technique could be applied to PCR testing of neurotropic viruses. Because CSF specimens used for PCR testing are generally limited to 1 mL or less, it is difficult to evaluate the usefulness of PCR testing with ultrafiltration due to the shortage of residual samples. We believe that one of the values of this study is in validating the use of ultrafiltration before PCR by using larger volume of CSF specimens. Given that the current protocol concentrated JCV particles with a diameter of 35–40 nm [5], it is likely that ultrafiltration of CSF specimens could be applied to PCR assays for other neurotropic viruses with larger virions, such as those of the family *Herpesviridae*. Furthermore, 10-kDa pore size membrane may concentrate picornaviruses smaller than JCV. Using the standard procedure, JCV DNA was detected in the CSF specimens of seven of 20 patients who were suspected of having PML. The ultrafiltration of CSF was unnecessary for PCR testing in four of these cases (patients 1–4), because more than 100 copies of JCV were detected in the DNA extracts per reaction. By contrast, only 20 copies or less per reaction of JCV DNA were observed in the CSF specimens of another three JCV-positive individuals (patients 5–7), and the number of viral DNA copies detected by PCR was markedly increased by ultrafiltration. Moreover, the CSF sample of a patient shown to be negative for JCV with the standard procedure was found to be positive after the ultrafiltration procedure (patient 8). This patient had HIV infection and had received combination antiretroviral therapy for more than 1 month. It is possible that the JCV DNA level in this patient’s CSF had been reduced by the combination antiretroviral therapy, as has previously been reported in cases of PML associated with HIV [21].

The detection limit of JCV DNA in the improved PCR for patients 1–7 was increased by introducing ultrafiltration. However, the calculated amounts of JCV per milliliter of the ultrafiltrated CSF were slightly lower than those of the non-treated specimens in five out of seven of these individuals (Table 1). The reason for this phenomenon is not clear at present and is under investigation. Since the JCV DNA copies in the flow-throughs of the CSF after centrifugation were less than 0.1% compared with those in the concentrates (data not shown), it is unlikely that any JCV particles passed through the ultrafiltration filter. Although the sensitivity of PCR assay was improved by introducing ultrafiltration, however, it remains possible that a proportion of the JCV particles could bind to filtration membranes, depending on the state of the specimen.

The significance of this study is not only in the potential to improve JCV testing in the CSF but also in the potential to unify PCR results with clinical data. All eight patients in whom JCV was detected in the CSF by PCR (patients 1–8) were diagnosed with PML based on neurological features and brain MRI findings. The individuals who were negative for JCV on CSF testing (patients 9–14 and 16–20) have also not been diagnosed with PML since this study except patient 15 in whom CSF JCV was detected more than 2 months later. It should be noted that many of these JCV-negative cases had atypical features, such as bilateral symmetric brain lesions, an absence of underlying disease, or both. The CSF specimens from these patients were negative for JCV in both the standard and ultrafiltrated procedures, making diagnosis unlikely. Overall, these observations indicate that the enrichment of viral particles and proteins by ultrafiltration does not lead to artificial or non-specific amplification during real-time PCR.

The ultrafiltration procedure used in this study improved routine PCR testing, especially when extremely low copy numbers of JCV DNA were present. However, the possibility that the highly sensitive PCR assay detects a persistently infecting JCV in the absence of PML cannot be totally excluded. Therefore, this technique is recommended to be used for the diagnosis in combination with the sequence analyses of mutations within the viral genome, which is characteristic of pathogenic JCV in patients with PML [21, 30, 31]. It will be interesting to compare the performance of PCR testing of ultrafiltrated CSF to that of brain biopsy. This improved PCR testing procedure may be particularly valuable when brain biopsy is difficult to perform or when a sufficient sample of brain tissue cannot be obtained. Further evaluation is in progress to determine the diagnostic value of PCR testing with ultrafiltration in a larger sample size of patients with PML.

## Conclusions

In conclusion, the usefulness of the real-time PCR testing of the CSF for JCV DNA can be improved by centrifugal ultrafiltration to enrich viral particle levels. The protocol described in this report is relatively simple and should serve as a useful tool for PML diagnosis when small amounts of JCV are released into the CSF.

## Data Availability

The analyzed datasets are available from the corresponding author on reasonable request.

## Abbreviations

CSF: Cerebrospinal fluid
DPBS: Dulbecco’s phosphate-buffered saline
FLAIR: Fluid-attenuated inversion recovery
HIV: Human immunodeficiency virus
JCV: JC virus
MRI: Magnetic resonance imaging
NIID: National Institute of Infectious Diseases
PCR: Polymerase chain reaction
PML: Progressive multifocal leukoencephalopathy
